# Engaging with community-based public and private mid-level providers for promoting the use of modern contraceptive methods in rural Pakistan: results from two innovative birth spacing interventions

**DOI:** 10.1186/s12978-016-0145-9

**Published:** 2016-03-17

**Authors:** Syed Khurram Azmat, Waqas Hameed, Hasan Bin Hamza, Ghulam Mustafa, Muhammad Ishaque, Ghazunfer Abbas, Omar Farooq Khan, Jamshaid Asghar, Erik Munroe, Safdar Ali, Wajahat Hussain, Sajid Ali, Aftab Ahmed, Moazzam Ali, Marleen Temmerman

**Affiliations:** 1 Department of Urogynecology, University of Ghent, Ghent, Belgium; 2 The Hospital for Sick Children, Toronto, Ontario Canada; 3 Marie Stopes Society, Research, Monitoring and Evaluation Department, Technical Services, Karachi, Sindh Pakistan; 4 Freelance Public Health Professional, Adelaide, South Australia Australia; 5 Research, Monitoring and Evaluation Department, Marie Stopes International, London, UK; 6 Department of Reproductive Health and Research, World Health Organization, Geneva, Switzerland

**Keywords:** Family planning, Birth spacing, Contraception, Vouchers, Community midwives, Suraj, Rural Pakistan

## Abstract

**Background:**

Family planning (FP) interventions aimed at reducing population growth have negligible during the last two decades in Pakistan. Innovative FP interventions that help reduce the growing population burden are the need of the hour. Marie Stopes Society - Pakistan implemented an operational research project - ‘Evidence for Innovating to Save Lives’, to explore effective and viable intervention models that can promote healthy timing and spacing of pregnancy in rural and under-served communities of Sindh, Punjab and Khyber Pakhtunkhwa provinces of Pakistan.

**Methods:**

We conducted a quasi-experimental (pre - and post-intervention with control arm) study to assess the effectiveness of each of the two intervention models, 1) *Suraj* model (meaning ‘Sun’ in English), which uses social franchises (SF) along with a demand-side financing (DSF) approach using free vouchers, and 2) Community Midwife (CMW) model, in promoting the use of modern contraceptive methods compared to respective controls.

Baseline and endline cross-sectional household surveys were conducted, 24 months apart, by recruiting 5566 and 6316 married women of reproductive age (MWRA) respectively. We used Stata® version 8 to report the net effect of interventions on outcome indicators using difference-in-differences analysis. Multivariate Cox proportional hazard regression analysis was used to assess the net effect of the intervention on current contraceptive use, keeping time constant and adjusting for other variables in the model.

**Results:**

The Suraj model was effective in significantly increasing awareness about FP methods among MWRA by 14 % percentage points, current contraceptive use by 5 % percentage points and long term modern method - intrauterine device (IUD) use by 6 % percentage points. The CMW model significantly increased contraceptive awareness by 28 % percentage points, ever use of contraceptives by 7 % percentage points and, IUD use by 3 % percentage points. Additionally the *Suraj* intervention led to a 35 % greater prevalence (prevalence ratio: 1.35, 95 % CI: 1.22–1.50) of contraceptive use among MWRA.

**Conclusion:**

*Suraj* intervention highlights the importance of embedding subsidized FP services within the communities of the beneficiaries. The outcomes of the CMW intervention also improved the use of long-term contraceptives. These findings indicate the necessity of designing and implementing FP initiatives involving local mid-level providers to expand contraceptive coverage in under-served areas.

## Background

Population growth in Pakistan presents significant challenges. Contraceptive prevalence rates (CPR) and fertility rates have largely remained unchanged, or have shown slow and insufficient improvements, during the last two decades [1]. Currently Pakistan has an estimated population of over 190 million people [2] and is the sixth most populous country [2, 3]. A high burden of population in developing countries with limited resources such as Pakistan makes resource allocation to health and development all the more difficult in the presence of other competing necessities [3, 4]. The challenge of high population growth in Pakistan necessitates the use and deployment of innovative plans that are effective in curtailing the future increase in population.

There is a rural–urban differential in key fertility and family planning (FP) indicators such as in Pakistan. The total fertility rate (TFR) is high recorded at 3.8 births per woman between 2010 and 2012 [5]. Urban–rural stratification indicates the TFR in rural areas (4.2 births woman) to be considerably higher than in urban areas (3.2 births woman) [5]. Additionally, the Pakistan Demographic and Health Survey (PDHS) 2012–13 reports a current CPR of 35 % for all contraceptive methods and a CPR of 26 % for modern method use with an urban (44.8 %) and rural (30.7 %) differential of 1.5 fold [5]. A high TFR combined with traditionally low CPR levels have resulted in a high unmet need for contraception in Pakistan [6] indicated by 20 % of currently married women of reproductive age (15–49) who desire to delay or limit their next birth [5].

The penetration of FP interventions in rural areas has remained lower compared to urban setting(s) demonstrated by the higher TFR and unmet need in rural areas [7]. With close to 63 % of the population living in rural areas in Pakistan [5, 8], there is considerable room for introducing FP interventions in targeted rural communities.

### Context

The World Health Organization (WHO) recommends engaging the private sector in FP promotion, considering its role in health care, including reproductive health (RH) service delivery in most settings [9]. In Pakistan’s context, less than half (45 %) of FP service provision through the public sector means that the private sector is meeting a significant proportion of contraceptive demand in the country [10]. However, the involvement of the private sector in FP promotion and delivery, although desirable, has limitations. The price and quality of family planning products – especially long term products, vary and are a constraint for potential FP method users in low income countries [9].

The World Health Organization (WHO) has suggested that, in order to overcome the lack of contraceptive services in regions of the world, the implementation of contracting out, social franchising and voucher schemes are of value [9]. Social franchising (SF) in combination with demand-side financing (DSF) based free voucher is an approach advocated to overcome financial constraints in order to increase access to, and uptake of, FP services [11]. Social franchises are mid-level private-provider networks and are considered to be effective business models having the potential to rapidly expand health services, promote access and contribute to national health goals [9].

Integration of FP service provision with existing public sector health service delivery mechanisms at the community level is an alternative approach, aiming to increase FP access and uptake for underserved communities. The National, Maternal, Newborn and Child Health (MNCH) program of Pakistan aims to improve MNCH indicators by deploying community based health workers known as Community Midwives (CMWs) [12]. CMWs are selected from communities they are most likely to stay in and work [10]. These CMWs are trained to provide individualized care to pregnant women, monitor their physical, emotional and social well-being, taking appropriate action within available resources, providing guidance to community members about maternal health issues, identifying conditions necessitating referrals and making those referrals to relevant practitioner [10]. The Lady Health Worker (LHW) program was assigned a parallel function role with the MNCH led CMW program or in other words the LHWs role was expanded in order to make child birth/delivery referrals for CMWs as well as to facilitate use and support of FP services by women in their catchment areas [12]. However, the available evidence suggested that the CMW program had difficulties in showing to show significant improvements in maternal health indicators due to weak linkages between these two programs [12]. Integration of FP service provision with existing CMW-provided reproductive health services can possibly ensure a continuum of care for the recipients. The training of CMWs and their close proximity to women has the potential to improve contraceptive access and uptake.

Enhancing the availability of these products and services in underserved areas is essential to improving national level FP indicators such as contraceptive prevalence rate including modern contraceptive uptake and reducing unmet need. The delivery of the products and services has brought about improvements in FP method uptake in urban settings [5]. It is essential to devise ways and means that address this problem in rural, hard to reach and underserved areas.

In order to produce evidence-based learnings for policy and practice for Maternal and Newborn Health in Pakistan, the Department of International Development (DFID), British Government and Australian Agency for International Development (AusAID) jointly funded a Maternal and Newborn Health Programme in Pakistan called ‘Research and Advocacy Fund (RAF)’ with a central objective for “Improved practices and supporting policies related to MNH affecting poor and marginalized people in Pakistan. The objective was to be achieved through large and small grants for Research and Advocacy in MNH; thereby linking evidence to policy and practice” [13].

Under this initiative, two intervention models were designed by Marie Stopes Society Pakistan as research initiatives for the RAF funding namely - 1) Social Franchising in combination with demand-side financing led voucher schemes, and 2) integration of FP services ong-term in particular with existing reproductive health services provided by community midwives - CMWs at the community level present an opportunity to test interventions aimed at improving FP indicators in hard to access remote areas [14, 15]. Recent evidence, from Pakistan, shows that family planning interventions, incorporating social franchising in combination with voucher scheme, have been instrumental in raising awareness and enhancing the use of intrauterine devices (IUDs) in study areas [10]. Prior to recommending a similar scaling up of this approach at the national level, given the variation in social and health seeking practices in different geographical areas of Pakistan, it was important to assess whether these findings are replicable in other districts also. In this context, therefore Marie Stopes Society (MSS) - Pakistan, implemented a 41-month (including 24 months of intervention) operations/operational research project titled ‘Evidence for Innovating to Save Lives’ [14–17]. The project’s aim was to explore effective and viable intervention models to promote healthy timing and spacing of pregnancies in rural and under-served communities of Sindh, Punjab and Khyber Pakhtunkhwa (KP) provinces in Pakistan [14–17].

### Objectives of the research project

The study was conducted to 1) to assess and compare the effectiveness of an intervention model, a private provider partnership i.e. Suraj social franchise model, with a control group, and 2) to assess and compare the effectiveness of an intervention model, FP integration in the existing MNCH services provided by Community midwives intervention model, with a control group, in promoting the use of modern contraceptive methods.

**Box 1:** **Primary and secondary outcomes**

Intervention	Primary outcome	Secondary outcome
*Suraj* model	Uptake of modern contraceptive methods	Awareness of contraceptive methods
Community Midwives model	Uptake of modern contraceptive methods	Awareness of contraceptive methods

### Intervention description

#### a) Study setting: Intervention and control arms

The project investigators employed a quasi-experimental (pre and post intervention with control) mixed method research study with sequential implementation at design level [14, 17]. The overall study design comprises of two (02) Qualitative and two (02) Quantitative data collection components or surveys.

**Fig. 1:**
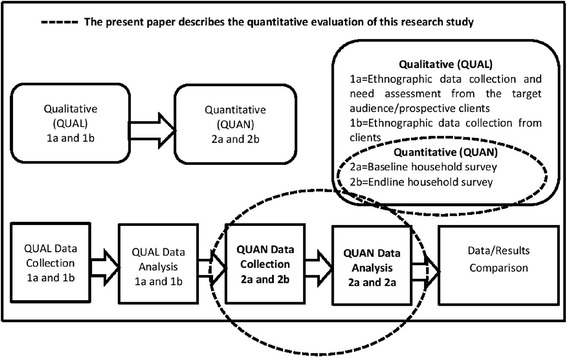
Overall study design flow chart

Hence, the present paper only describes the Quantitative 2a and 2b surveys i.e. the Baseline and Endline comparison on selected indicators as presented in Fig. 1 below.

Kindly refer to the below study design flow chart as Fig. 1 to understand the study components as the present paper describes the quantitative baseline and endline results:

The study was conducted in eight districts of Sindh, Punjab and KP provinces of Pakistan. Within the districts, Marie Stopes identified rural and under-served Union Councils (UCs) for inclusion in the study. Districts were selected based on key socioeconomic, demographic and reproductive health indicators (Table 1). Interventions were purposefully allocated: in Sindh, district Naushero Feroze was selected as an intervention (*Suraj* model) district and Nawabshah as a control district. In Punjab province, districts Pakpattan and Rajanpur were selected as intervention districts for the CMW model while district Khanewal was identified as intervention district for *Suraj* model whereas district Bahawalpur served as the control district. For KP, district Haripur served as an intervention (*Suraj* model) district while district Abbottabad was the control.

**Table 1:** Comparability of intervention and control districts

Indicators	Province							
	Sindh		Lower Punjab				Khyber-Pakhtunkhwa	
	Intervention	Control	Intervention			Control	Intervention	Control
	Naushero Feroze (Suraj)	Nawabshah	Khanewal (Suraj)	Pakpattan (CMW)	Rajanpur (CMW)	Bahawalpur	Haripur (Suraj)	Abbottabad
Estimated population size	1,087,571	1,071,533	1,286,680	2,068,490	1,103,618	2,433,091	692,228	880,666
% of female Pop. Age 15–49	22.2	22.6	21.9	22	20.2	21.4	23.8	23.7
CPR (modern method)	20.8 %	15.4 %	17 %	19 %	11 %	24 %	29.7 %	29.1 %
% literate	39.1	34.1	49	42	34	37	53.7	56.6
% of households with electricity	69.3	75.5	68	76	59	50	76.3	75
% of households with access to potable water	23.6	23.6	12	15	8.5	16	49.6	29.9
No. of UCs	51	50	101	64	47	108	45	46

#### *b) Suraj* model - intervention arm

MSS established a private health providers’ network branded as ‘*Suraj*’ (meaning ‘Sun’ in English) in the intervention districts [10]. The model is a partnership between MSS and private local health service providers (mainly mid-level) for the provision of quality contraceptive services. Ten *Suraj* providers per district were selected. Each *Suraj* provider operated a health care facility, covering a population ranging from 12–16,000 that resided within a 3–4 km radius around the heath facility. The *Suraj* providers were located at an average distance of 40–50 km from District Head Quarter (DHQ) hospitals. In order to minimize any spill-over effect between areas of *Suraj* providers, it was ensured that the minimum distance between two providers was large enough.

The selection and training of *Suraj* providers was a three step process. First, mapping of districts was conducted to ascertain the existing number of health care facilities and providers in a given district. Second, providers were selected for training by arranging individual meetings with MSS field teams and collection of information on provider eligibility criteria (see Table 2) [10]. For the details of *Suraj* intervention components, refer to Table 4.

**Table 2:** Provider eligibility criteria - *Suraj* intervention model

• Provider should be female (preferably married) aged 18–35 • Preference was for non-MNCH midwives, however if none were available LHVs or nurses were considered for inclusion • Provider had at least ten years of education, preferably with science subjects • Preference if provider was a native and practicing in the same area • Had more than two years of work experience in FP/RH • Provider was willing for partnership, expansion of quality services and business • Provider was willing to be checked for her work, report, audit etc. • Provider was willing to provide the physical infrastructure to meet the basic needs of a standard FP service Centre such as privacy for clients, proper place for examination, waiting area, enough ventilation and light arrangement and a sterilization area	

Third, *Suraj* SF providers were imparted training to improve their skills for provision of quality FP services, and enable them to look after the business side of their ventures.

#### c) CMW model - intervention arm

In contrast to the *Suraj* model, the community midwives - CMW intervention model was an arrangement between MSS and CMWs for the provision of quality contraceptive services in the community. We obtained a list of CMWs from the Maternal Newborn and child Health (MNCH) program and ten CMW providers for each district were selected. Each CMW provider covered a population ranging from 7000 to 12,000 that resided within a 3–4 km radius around the facility which is operated by a provider. The CMW providers were located at an average distance of 40–70 km from the district headquarter hospital. The selection of CMW ensured a minimum distance between any two CMW providers in order to minimize any spill-over effects. The selection and training of CMW providers was also a three step process similar to that adopted for *Suraj* providers CMW provider eligibility criteria are listed here (Table 3). For the details of CMW intervention components, refer to Table 4.

**Table 3:** Provider eligibility criteria - CMW intervention model

• Permanent resident of rural areas • Female, preferably married between 18–35 years of age • Had at least ten years of education, preferably with science subjects • Experience of working in the community • Certification with Pakistan Nursing Council (PNC) and registered with MNCH • Willing for partnership, expansion of quality services and business • Willing to be checked for her work, report, audit etc. • Willing to provide services on standardized rates

**Table 4:** Intervention components

	Intervention items	Description	Inclusion in Suraj model	Inclusion in CMW model	Control
1	Training on reproductive health/family planning and post training evaluation	Medical: reproductive health and family planning, counselling, quality of services, and IUD insertion and removal; Business: basic budgeting skills, record keeping, stock management, branding (excluded for CMW), marketing, and the voucher management (excluded for CMW). The training was followed by post training evaluation conducted by an external consultant (a senior medical doctor).	Yes	Yes	No
2	Female community mobilizer (FCM)	An FCM was also a local resident of the community; she underwent training on FP methods, voucher distribution system, and data recording. She paid door to door visits, raised awareness, generated referrals and distributed vouchers for the IUD to eligible women, identified through poverty scale.	Yes	Yes	No
	*Each service provider was complemented with one FCM*				
3	Male community mobilizers (MCM)	An MCM was a local resident of the district; he underwent training and was responsible to target male community members. He formed community support groups which comprised key community stakeholders and conducted frequent Mohallah (locality) meetings	Yes	Yes	No
	*There is one MCM per 10 service providers in a district*				
4	Voucher for long- term contraceptive method (Intra- uterine device)	A voucher was worth PK Rs 200 (US$2.27) and only for IUD (insertion, follow-up and removal). A voucher may be redeemed at Suraj clinic; later the reimbursement was sent to the provider against her claim. Free voucher provision was based on a wealth based poverty assessment tool which was managed by the Field Community Mobilizers in the field before distributing vouchers. The said tool ask questions about wealth status including household structure, number of household members, number of meals, number of dependent members, sanitation, access to reproductive health services, daily household income, source of fuel used for cooking, source of drinking water. Clients received a voucher if their score fell between the minimum score of 9 and 20 (inclusive) on a scale of 27.	Yes	No	No
5	Branding/Marketing	Providers were branded ‘Suraj’ clinics while marketing was done through FCMs, posters, wall paintings, leaflets, etc. The ‘Suraj’ logo was displayed prominently in Urdu outside all clinics.	Yes	No	No

#### d) Control arm

The recruitment for providers in control districts was a three step process. First, mapping was initiated to get information on the existing number of health care and family planning (FP) facilities and providers in terms of distance and accessibility to women. Second, an MSS team comprising district and regional personnel identified the Union Councils (UCs) based on locally available records. Within each Union Council an MSS team member met with different key stakeholders such as pharmacists, drug stores, UC Mayors, farmer-councilor, community based organizations, influential personalities and others to capture key information on population, location of private providers, Union Council boundaries, number of schools, male and female literacy, number of healthcare centers’ such as basic health units, rural health centers and tertiary care hospitals. Third, a series of meetings with each provider/facility was conducted to invite the providers for participation in the study. Providers were considered eligible for participation provided the following criteria were met:

a) Health facility owned or staffed by a female; b) provider lived in the same community; c) provider was interested in providing family planning services; d) provider must have formal medical qualifications; e) there must be adequate facility infrastructure (e.g. space to perform family planning services, availability of required instruments/equipment and essential amenities such as running water and electricity, and sanitation and waste disposal facilities); and f) provider must be willing to adhere to the study protocol for control sites (i.e. record keeping and reporting).

The providers in the control arm were not given any exposure to study interventions. A total of 3 Rural, 10 Basic centers and 14 CMWs were recruited for this study. Each facility/provider was located approximately 30 km away (in any direction) from the district headquarter hospital in the predominantly rural area and covered a population ranging between 8000-12000 for CMWs and 35–40,000 for basic and rural health centers.

The minimum distance between any two facilities/providers was large enough to avoid a spill over effect. For the details of intervention components, refer to Table 4.

## Methods

### Study duration

As mentioned earlier, the research project was a 41 months initiative commencing in October, 2010 and ending in March, 2014 including 24 months of intervention (i.e. service provision) [14, 17].

### Endline evaluation study design

Pre (baseline) - and post-intervention (endline) cross-sectional surveys were conducted to assess the impact of interventions on the use of modern contraceptive methods. A baseline household survey was conducted prior to the implementation of the interventions (a benchmark for future evaluations of the project’s key performance indicators). Towards the end of project interventions, an endline cross-sectional household survey was conducted to gauge the impact of the two interventions by measuring the same set of indicators including reproductive health and family planning Awareness, behavior and practices of the respondents.

#### a) Study participants

At the baseline, married women of reproductive age (MWRA) between 15–49 years of age with at least one child less than 2 years of age were included in the study and interviewed. The endline survey included two groups of MWRA, 1) MWRA between 15–49 years of age and with at least one child less than 2 years of age and 2) MWRA between 15–49 years of age irrespective of the number of children. MWRA who were mentally or physically handicapped and were unable to give an interview, or who refused to provide informed consent or were unmarried/separated/widowed were excluded.

#### b) Sample size

The overall sample size for baseline survey was 5566 comprising of 1995, 1435 and 2136 MWRAs recruited from the Suraj, community midwives (CMW) and control catchment areas respectively. For baseline a minimum of 70 interviews were conducted per cluster or service provider catchment area. For endline, sample size calculations were run separately for two groups based on anticipated change in CPR: 1) MWRA and 2) MWRA with a child under the age of 2 years. The key indicator (contraceptive prevalence rate - CPR) being assessed in each required a separate sample size calculation. Sample size was calculated for treatment groups rather than districts. The calculation is presented below:

$$ \begin{array}{l}n=\frac{deff\times {\scriptscriptstyle \frac{{\left({z}_{\alpha }+{z}_{\beta}\right)}^2\left({p}_1\left(1-{p}_1\right)+\left({p}_2\left(1-{p}_2\right)\right.\right)}{\delta^2}}}{\left(1-l\right)}\\ {}n=\frac{2\times {\scriptscriptstyle \frac{{\left(2.241+0.842\right)}^2\left(0.303\left(1-0.303\right)+\left(0.403\left(1-0.403\right)\right.\right)}{0.1^2}}}{\left(1-0.1\right)}\end{array} $$

The sample size for MWRAs with young children was based on a comparison between odds ratios. We took the most conservative measure, adjusted for a pooled *p* of 0.05, that resulted in non-overlapping CIs based on a 10 % increase from the mean baseline modern CPR figures by intervention arms compared to a 2 % increase from mean baseline modern CPR in controls. Table 5 below shows the estimated sample for two different types of respondents by districts.

**Table 5:** Names of districts and number of interviews

	Sindh		Punjab				Khyber Pakhtoonkhwa		
Target respondents	Noshero Feroze (Suraj)	Nawabshah (control)	Pak Pattan (CMW)	Rajanpur (CMW)	Khanewal (Suraj)	Bahawalpur (Control)	Haripur (Suraj)		Abbotabad (control)
Mothers (currently married) having at least one <2 year child	380	380	570	570	380	380	380		380
Currently married women of reproductive age 15-49 years	320	320	480	480	320	320	320		320
District wise Total	700	700	1050	1050	700	700	700	700	
Grand Total	6300								

#### c) Sampling strategy

We used probability proportional to population size (PPS) technique within each of the three study arms to select study participants. Each target area of study districts was considered as a separate stratum. The data collection was conducted within the same catchment population of the study sites for both the baseline and the endline surveys. Prior to data collection, all the households (within 4–5 km radius) around each selected healthcare facility were independently allotted a unique identifier. A list of households with unique identifiers in the intervention and control areas comprised the sampling frame of households which were selected using simple random techniques through statistical package for social sciences (SPSS) version 17.0. A household was considered as a primary sampling unit at both baseline and endline surveys. If more than one MWRA, meeting survey criteria, were identified in a randomly selected household, only the first one (or if she refused, then the next one) was recruited for data collection.

#### d) Data collection and management

The baseline data were collected during March-July 2011 while endline survey was conducted between July-August, 2013. We adapted the questionnaire from the Pakistan Demographic and Health Survey (PDHS) 2006–07 with modifications to measure use of any contraceptive methods. The questionnaire was designed to capture information on socio-demographic characteristics, awareness of reproductive health (RH) and family planning (FP), FP practices, and health seeking behaviours, health care access and FP needs of study participants. The questionnaires were translated into Urdu and pre-tested prior to commencement of data collection. Completed questionnaires were checked for completeness and logical errors. Reliability checks helped ensure that similar data were received. Principal and co-Investigators routinely visited field to ensure the quality of data. Forms were checked for logical errors, missing values, and unclear responses during those visits. All survey data were double-entered to ensure the quality of data and minimize entry and logical errors using a specifically designed data entry programme on FoxPro version 6.0.

#### e) Data analysis

We used SPSS software version 17.0™ to analyse the data and generate tables from a list of survey variables for descriptive analysis. The analysis was performed for MWRA with a child less than 2 years of age - a sub-group of the sample. This was done to ensure comparability with the baseline information collected on a similar group of MWRA.

Descriptive statistics were computed for socio-demographic variables and potential associated factors. Frequencies, proportions, means and standard deviations were obtained as appropriate. Where needed, continuous variables were categorized through important cut-off-points and variables such as total number of children, years of education and age were categorized based on commonly used categories.

Stata® version 8 was used to assess the effect of interventions (*Suraj* SF model vs. control and CMW model vs. control) on outcome indicators through Difference-In-Difference (DID) analysis. Univariate DIDs were estimated employing the following steps: a) at first, we calculated the change (from baseline to endline) in the control arm and the change (from baseline to endline) in the intervention arm; b) we then estimated the net effect of intervention by subtracting the change in control arm from the change in intervention arm. Similar procedure was followed for different key indicators.

We conducted multivariable analysis, to determine factors associated with current contraceptive use (dependent variable) in each intervention arm, using Cox proportional hazard regression keeping time constant adjusting for clusters. Prevalence ratio with 95 % confidence interval (CI) was computed for each independent variable by likelihood ratio test for significance of estimated regression coefficients. Variables with *p* < 0.25 on univariate analysis were considered for a stepwise multivariate analysis. Wald statistic and likelihood ratio test were used to assess the significance of variables and models respectively, towards obtaining a parsimonious and meaningful model. The analysis was adjusted for independent variables such as age, education, province, number of children and social economic status.

#### F: Ethics statement

Verbal and written (participants’ signature or thumb impression) informed consent were obtained from the study respondents. Personal identifiers were not recorded to ensure confidentiality. Designated authorized personnel had completed hard copies of the questionnaires under safe keeping. Electronic version of the data was stored on password protected computers. The project was approved by the Program Oversight Committee of Research and Advocacy Fund (RAF). The ethical approval for the research study was provided by the National Bioethics Committee (NBC) of Pakistan (Ref no: 4-87/10/NBC-43/RDC/).

Note: Brief information describing design and methods of study is also published in a separate paper/s [17].

## Results

We present findings for married women of reproductive age (MWRA), with a child less than 2 years of age, recruited at baseline (5566) and endline (2892).

### a) Socio demographic characteristics

Table 6 describes the socio demographic characteristics of MWRA. The mean age of MWRA at the baseline and endline was 28.0 ± 5.5 and 29.1 ± 5.6 respectively. The average marriage age (age at first marriage) of MWRA between the two time points was around 20 years. Illiteracy proportions for MWRA demonstrated a drop of 8 % points at the endline (Table 6). MWRA who reported working increased slightly by 2.7 % at the endline. A concomitant increase in unskilled employment and agricultural work by their husbands is also noted (Table 6) and might be explained by an increase in seasonal agricultural work.

**Table 6:** Socio-demographic characteristics of respondents

Characteristics	Baseline	Endline	*P-value (Baseline vs Endline)*	Endline		
	All participants 5566 (%)	All participants (*N* = 2892) (%)		*Suraj* Intervention (*N* = 1105) (%)	CMW Intervention (*N* = 712) (%)	Control Arm (*N* = 1075) (%)
Age of MWRA						
15–19 years	3.0	1.6	<0.0001	2.1	1.1	1.5
20–24 years	19.0	18.9	0.9114	17.5	18.4	20.7
25–29 years	33.0	33.8	0.4589	34.8	27.1	37.2
30–34 years	27.0	25.3	0.0926	26.4	30.3	20.9
35–39 years	14.0	15.5	0.0632	16.1	16.0	14.6
40–44 years	3.0	4.2	0.0039	2.6	6.3	4.5
45–49 years	1.0	0.6	0.0593	0.5	0.7	0.7
Mean age ± SD (median) years	28.0 ± 5.5 (28)	29.1 ± 5.6 (30)	<0.0001	28.8 ± 5.3 (28)	29.9 ± 5.9 (30)	28.8 ± 5.6 (28)
Age at time of marriage (mean ± SD)	20 ± 3.4	19.8 ± 3.3	0.009	20.3 ± 3.2	19.5 ± 3.5	19.6 ± 3.4
Ethnicity						
Urdu	7.0	5.5	0.0079	8.9	2.7	3.9
Sindhi	15.0	17.8	0.0009	23.6	0.3	23.4
Punjabi	29.0	26.5	0.0153	34.5	25.0	19.2
Hindco	22.0	19.1	0.0019	22.4	0.6	27.8
Saraiki	24.0	29.0	<0.0001	7.3	71.3	23.3
Others (Pashto, Kashmiri, Balochi)	2.9	2.2	0.0578	3.3	0.1	2.3
Education status of the MWRA						
Illiterate	55.9	48.0	<0.0001	40.4	66.4	43.5
Can read or write only/less than 1 class	1.6	3.8	<0.0001	3.1	5.3	3.4
Primary (1 to 5)	15.5	16.0	0.5485	17.9	10.7	17.7
Middle (6 to 8)	7.7	9.4	0.0071	11.2	6.2	9.7
Matriculation and Higher	19.3	22.8	0.0002	27.4	11.5	25.7
Employment status of the MWRA						
Housewife	93.1	90.4	<0.0001	95.6	83.3	89.8
Working	6.9	9.6	<0.0001	4.4	16.7	10.2
Employment status of Husbands						
Unemployed	4.6	2.9	0.0002	3.8	1.4	2.9
Skilled Employment	60.3	52.5	<0.0001	63.2	37.6	52.1
Unskilled Employment	27.9	30.1	0.0338	22.8	43.6	28.6
Agriculture/farming	7.0	14.6	<0.0001	10.2	18.8	16.4

### b) Contraceptive methods awareness

At the baseline, awareness was relatively lower in community midwives - CMW areas than Suraj areas; however, at endline awareness about pills, condoms, injectables, and IUDs increased to above 80 % in both the intervention arms, leading to a greater increase in overall awareness (from 61.3 % at baseline to 94.4 % at endline) in CMW areas than Suraj areas. In Suraj intervention areas, overall awareness about contraceptive methods improved from the baseline (77.6 %) to endline (97.6 %) (*p* < 0.001). The largest increase in awareness levels was reported for Intra Uterine Devices - IUDs (absolute percentage change: 29.2 %) followed by contraceptive pills (absolute percentage change: 25.2 %). Male sterilization and implants were the least known methods across the three study groups (Table 7).

**Table 7:** Contraceptive method awareness among MWRA

Characteristics	Baseline			Endline		
	*Suraj* Intervention (*N* = 1995) (%)	CMW Intervention (*N* = 1435) (%)	Control Arm (*N* = 2136) (%)	*Suraj* Intervention (*N* = 1095) (%)	CMW Intervention (*N* = 712) (%)	Control Arm (*N* = 1075) (%)
Awareness of contraceptive method						
Any contraceptive method	77.6	61.3	88.3	97.6	94.4	93.9
Pills	68.7	45.9	80.4	93.8	88.6	87.0
IUDs	54.4	28.6	55.3	83.6	82.3	74.0
Injectables	65.1	42.4	73.9	89.0	86.1	83.3
Implant	15.4	11.2	20.1	36.6	43.1	38.5
Condom	60.4	34.9	66.0	76.7	72.2	80.7
Female Sterilization	51.1	26.0	52.9	72.2	75.3	78.0
Male Sterilization	30.6	17.7	29.4	28.9	44.9	37.6
Withdrawal	36.7	14.1	36.4	42.4	49.6	51.9
Periodic abstinence	36.9	15.5	39.9	39.5	48.6	47.3
Awareness of where to get contraceptives						
Any modern method	67.8	43.1	76.5	96.5	89.6	88.7
Pill	55.4	29.4	64.9	91.0	76.8	79.4
IUD	44.5	17.6	47.0	80.2	68.4	66.5
Injection	52.7	27.0	61.9	86.2	74.4	77.0
Implant	13.9	4.0	16.2	36.9	38.1	33.7
Condom	51.8	22.9	54.6	74.4	61.5	73.6
Female Sterilization	39.1	15.4	42.5	68.3	63.5	69.4
Male Sterilization	23.6	9.1	23.7	26.6	36.8	31.0

### c) Ever and current contraceptive use

Ever and current contraceptive use patterns for MWRA are described in Table 8. MWRA in the Suraj intervention arm reported a 13.7 % points increase in current contraceptive use from 34.0 % at baseline to 47.7 % at end line (*p* < 0.0001). Current contraceptive use in the CMW intervention arm increased from 17 % at baseline to 24.6 % at endline (*p* < 0.0001). Method mix indicates that the use of modern methods among the MWRA in the Suraj intervention model increased by 7.6 % (*p* < 0.0001) while it increased by 6.1 % (*p* < 0.0003) among MWRA in the CMW intervention model (Table 8).

**Table 8:** Ever and current contraceptive use reported by MWRA

Characteristics	*Suraj* Intervention			CMW Intervention			Control arm		
	Baseline (*N* = 1995) (%)	Endline (*N* = 1095) (%)	*p* value	Baseline (*N* = 1435) (%)	Endline (*N* = 712) (%)	*p* value	Baseline (*N* = 2136) (%)	Endline (*N* = 1075) (%)	*p* value
Ever use of contraception^a^									
Any method	49.9	65.2	<0.0001	30.2	41.9	<0.0001	48.3	53.2	0.0088
Pills	10.3	13.3	0.012	7	9.8	0.0238	8.6	10.3	0.1152
IUD	5.3	12.1	<0.0001	4.3	8.3	0.0002	5.8	5	0.3494
Injections	12.3	15.2	0.0233	7.2	14.5	<0.0001	10.1	12.1	0.0845
Condom	23.3	23.2	0.9498	8.2	8.7	0.6936	19.6	21.1	0.3169
Implant	0.4	0.9	0.0793	0.2	1	0.0189	0.2	0.7	0.0265
Female Sterilization	4.3	3.6	0.3452	2.1	3.1	0.1567	3.4	5	0.0278
Male Sterilization	0.2	0.4	0.4652	0.6	0.3	0.1799	0.1	0.7	0.0035
Traditional Method^b^	14.5	19.9	0.0001	7.4	12.1	0.0003	15.5	22.1	<0.0001
Current use of contraceptive^c^	34	47.7	<0.0001	17.1	24.6	<0.0001	26.9	35.2	<0.0001
Any Modern method	30	37.6	<0.0001	14	20.1	0.0003	24.1	28.7	0.0048
Pills	3.8	5.1	0.087	3.1	2.5	0.4355	2.9	3.7	0.2219
IUD	3	8.3	<0.0001	2.1	4.1	0.0078	3.3	3.4	0.8816
Injections	6.3	6.7	0.6648	3.7	5.3	0.0829	5.1	5.6	0.5495
Condom	13.8	13.5	0.8165	3.6	4.9	0.1493	9.6	11	0.2134
Implant	0.3	0.5	0.3817	0.1	0.8	0.0066	0.3	0.4	0.7401
Female Sterilization	2.9	3.4	0.4414	1.2	2.4	0.0368	2.9	4.5	0.0188
Male Sterilization	0	0.2	0.1255	0.2	0	0.5533	0	0.1	0.3348
Traditional method^b^	3.9	10.2	<0.0001	3	4.5	0.0749	2.8	6.5	<0.0001

^a^multiple responses

^b^Traditional Methods include withdrawal, abstinence and Lactation Amenorrhea Method (LAM)

^c^current contraceptive use was asked from all MWRA

### d) Impact analysis

#### *Suraj* intervention versus control

At the endline, the CPR of the Suraj intervention group was 48 % and resulted in a net CPR increase of 5 % (*p* < 0.05) (Table 9). This net increase in CPR among MWRA of the *Suraj* intervention model can be explained by the significantly increased use of IUDs (6 %) (*p* < 0.001), and a concomitant significant reduction in use of withdrawal method (−1 % *p* < 0.001) and condoms (−3 % *p* < 0.001) (Table 9). A net increase of 14 % (*p* < 0.01) among MWRA who had heard about contraception demonstrates positive impact of the *Suraj* intervention model on contraceptive awareness (Table 9).

**Table 9:** Difference-in-difference results for key indicators between *Suraj* intervention arm and control arm

	Control		*Suraj* Intervention		Absolute difference (% change)^a^		Net effect (% change)^b^
Indicators	Baseline (%)	Endline (%)	Baseline (%)	Endline (%)	Control	*Suraj*	
Ever use of any contraceptive	48	53	50	65	5	15	10***
Ever heard of any contraceptive	88	94	78	98	6	20	14***
Current use of any contraceptive	27	35	34	48	8	13	5*
Current contraceptive method of choice							
Any Modern method	24	28	30	38	4	8	4
Pill	3	4	4	5	1	1	1
IUD	3	3	3	8	<1	5	6***
Injections	5	6	6	7	1	1	<1
Condom	2	6	3	4	4	1	−3***
Withdrawal	10	11	14	14	1	0	−1

*P*-value: ***p < 0.01; **p < 0.05; *p < 0.1

^a^Absolute difference is the percentage change from baseline to endline

^b^Net effect is the percentage change in intervention group adjusting for the percentage change in control group

### Community midwives - CMW intervention versus control

The results demonstrate a significant positive effect of CMW intervention on contraceptive awareness, ever use, and use of modern long term contraceptive methods such as IUDs (Table 10). The net CPR in the CMW areas remained unchanged from the baseline to the endline (Table 10). However, modern methods usage showed a net significant increase of 3 % in IUD use. Additionally, a net decrease of 4 % in withdrawal usage was also observed in CMW intervention areas (Table 10). A similar positive effect of CMW intervention was observed on contraceptive awareness which increased by a net 28 % (*p* < 0.001).

**Table 10:** Difference-in-difference results for key indicators between CMW intervention arm and control arm

	Control		CMW Intervention		Absolute difference (% change)^a^		Net effect (% change)^b^
	Baseline (%)	Endline (%)	Baseline (%)	Endline (%)	Control	CMW	
Ever use of any contraceptive	48	53	30	42	5	12	7**
Ever heard of any contraceptive	88	94	61	94	6	33	28**
Current use of any contraceptive	27	35	17	25	8	8	0
Current contraceptive method of choice							
Any Modern method	24	28	14	20	4	6	2
Condom	10	11	4	5	1	1	0
Pill	3	4	3	3	1	0	−1
IUD	3	3	2	5	0	3	3**
Injection	5	6	4	5	1	2	1
Withdrawal	2	6	2	3	4	1	−4***

*P*-value: ***p < 0.01; **p < 0.05; *p < 0.1

^a^Absolute difference is the percentage change from baseline to endline

^b^Net effect is the percentage change in intervention group adjusting for the percentage change in control group

### e) Factors associated with current contraceptive use

#### *Suraj* intervention model

MWRA in *Suraj* intervention arm had a 35 % greater prevalence (prevalence ratio (PR): 1.35, 95 % Confidence interval (CI): 1.22–1.50) of current contraceptive use compared to their counterparts in the control arm while adjusting for other factors (Table 11). Older MWRA (35+ years, PR: 1.21, 95 % CI:1.05–1.39), those with education (1–8 years [PR: 1.22, 95 % CI:1.07–1.38] and secondary to higher [PR: 1.43, 95 % CI: 1.26–1.62]) and greater number of children (3–4 [PR: 1.42, 95 % CI: 1.26–1.60] and 5+ [PR: 1.54, 95 % CI: 1.34–1.76]) were more likely to use contraception than their counterparts in the control arm (Table 11).

**Table 11:** Multivariate-Cox proportional hazard analysis of factors associated with current contraceptive use among married women with at least one child < 2 years in *Suraj* intervention and control areas across Pakistan

Variables	Current contraceptive use		
	Prevalence ratio	95 % CI	*P* Value
*Suraj* Intervention < is this an interaction term?>			
Control	1	-	-
Intervention	1.35	1.22–1.50	<0.001
Education			
Illiterate	1	-	-
Class 1 to 8	1.22	1.07–1.38	0.002
Secondary and higher	1.43	1.26–1.62	<0.001
Province			
Sindh	1	-	-
Punjab	0.90	0.80–1.02	0.094
KPK	0.87	0.76–0.99	0.039
Total children			
0–2	1	-	-
3–4	1.42	1.26–1.60	<0.001
5+	1.54	1.34–1.76	<0.001
Socio-economic Status			
Lowest SES	1	-	-
Middle SES	1.54	1.33–1.77	0.000
Highest SES	1.97	1.72–2.26	0.000
Age in years			
<25	1	-	
>25 to < = 30	0.99	0.89–1.13	0.983
>30 to < =35	1.12	0.95–1.31	0.171
>35	1.21	1.05–1.39	0.009

### Community midwives - CMW intervention model

MWRA aged more than 35 years had significantly increased prevalence ratios of current contraceptive use (PR: 1.36, 95 % CI: 1.07–1.72), followed by MWRA aged 31 to 35 years (PR: 1.25, 95 % CI: 1.00–1.54). The CPR showed significant increase with higher levels of education i.e. current contraceptive use was highest among MWRA having secondary or higher education (PR: 2.26, 95 % CI: 1.91–2.66) followed by MWRA with 1–8 years of education (PR: 1.86, 95 % CI: 1.59–2.18) compared to those without any education. MWRA with 5 or more children had significant increase in current contraceptive use (PR: 1.81, 95 % CI: 1.45–2.27) as compared to MWRA having no children or up to 2 children (Table 12).

**Table 12:** Multivariate-Cox proportional hazard analysis of factors associated with current contraceptive use among married women with at least one child < 2 years in CMW intervention and control areas across Pakistan

Variable	Current contraceptive use		
	Prevalence ratio	95 % CI	*P* value
Age in years			
<25	1	-	-
>25 to < = 30	0.98	0.80–1.18	0.812
>30 to < =35	1.25	1.00–1.54	0.044
>35	1.36	1.07–1.72	0.012
Education			
Illiterate	1	-	-
Class 1 to 8	1.86	1.59–2.18	<0.001
Secondary and higher	2.26	1.91–2.66	<0.001
Total children			
0–2	1	-	-
3–4	1.86	1.53–2.25	<0.001
5+	1.81	1.45–2.27	<0.001
CMW Intervention			
Control arm	1	-	-
CMW arm	1.00	0.81–1.24	0.952

## Discussion

Findings of this quasi-experimental study demonstrate that both FP intervention models, i.e. the *Suraj* SF model along with demand-side financing vouchers, and the integrated FP services with existing CMW providers model at the community level, are effective in improving key FP indicators such as women’s awareness of FP methods, ever use and current use of contraceptives besides the use of long term contraceptives such as IUDs, in hard to reach remote areas of Pakistan.

Our findings indicate that the *Suraj* SF intervention model was instrumental in eliciting a significant net increase of about 14 % (*p* < 0.001) in awareness about FP methods among MWRA. Previous evidence from Pakistan shows that FP interventions, such as social franchising incorporating FP service delivery through a voucher scheme, have been successful in raising FP awareness and the use of IUDs including improvements in the IUD continuation rates [10, 18, 19]. Recently in the year 2012 in Pakistan, a social franchising initiative along with free contraceptive vouchers significantly increased the awareness of modern contraceptives among women by 5 % in intervention areas [10]. Increase in CPR is reportedly rooted in increased Awareness levels of all contraceptive methods especially modern methods and places to obtain them [20]. Additionally, we found a net increase of 5 % (*p* < 0.001) in the current contraceptive use and 28.5 % increase in ever use of modern contraceptive. The results corroborate with the earlier study conducted in Pakistan in similar settings [10]. In our study IUD use among MWRA in the *Suraj* SF intervention recorded a net increase of 6 % (*p* < 0.001) similar to a significant increase of 11.1 % previously recorded in the uptake of IUDs by MWRA, which were being promoted with vouchers [10]. The significantly increased usage of IUDs in *Suraj* intervention areas may be explained by the accompanying decrease in the usage of traditional method of withdrawal. Regression analysis further identified that the *Suraj* SF intervention model led to a 35 % significantly greater prevalence of current contraceptive use among MWRA compared with control.

Current contraceptive use in *Suraj* intervention areas was also found to be associated with higher education, parity and socio-economic status. These observed changes indicate the effectiveness of *Suraj* interventions and reach of the program in increasing contraceptive use among MWRA in *Suraj* areas. From programmatic view point, an encouraging aspect of increased knowledge and awareness of MWRA about contraceptive methods is the successful awareness raising efforts by MSS Field Health Educators who were the agents of information in this regard. This is encouraging programmatically since it shows that enhancing the capacity and efficacy of frontline workers can greatly impact perceptions about contraception that ultimately translate into increased and consistent usage of these methods [21, 22]. Increased Awareness levels have also translated into the understanding and demand creation for contraception that is long term rather than shorter term methods indicated by enhanced IUD related Awareness and usage. The effectiveness of *Suraj* SF intervention model is a critical finding due to the importance of long term contraceptive use in ensuring desirable spacing between births and reducing unwanted pregnancies.

The CMW model was successful in several aspects. We found that the CMW intervention model had a significant positive effect on contraceptive awareness and ever use. The CMW intervention model increased the contraceptive awareness among MWRA by a net 28 % (*p* < 0.001) in the intervention arm from baseline to end line. The CMW model also resulted in an 8 % increase in CPR between baseline and endline. However, the net effect was nullified when CPR in the control arm was taken into account. We also found that while the CMW model did not significantly affect the overall CPR i.e. both traditional and modern methods combined, the model did significantly increase the use of long term contraceptive method - IUDs among MWRA by a net 3 % (*p* < 0.05). The method mix of modern contraceptives use highlights a shift towards long term contraceptive methods among MWRA in CMW areas. The CPR was associated with higher levels of education in the CMW intervention model where MWRA with secondary or higher education had a 2.26 times greater prevalence of current contraceptive use. Considering the greater prevalence of current contraceptive use among MWRA who have at least 3 children, are educated and older than 30 years in CMW intervention areas, it appears that these determinants are driving the 3 % net increase in IUD use in the CMW arm.

Among the two intervention arms, the *Suraj* intervention model showed the most encouraging results. The current contraceptive use was the highest at 48 %; a proportion that is exceptionally encouraging since it is 13 % higher than the national averages [5], pointing towards the effectiveness of strategies adopted through this intervention. Women were generally appreciative of the quality of counseling in managing side effects and resultant fewer clinic visits besides availability of free FP (IUD) services [19]. They highly valued cleanliness, privacy, and confidentiality, sterilization of instruments and ease of communication with *Suraj* providers [19]. On the provider side, IUD insertion and infection- prevention training have been reported to enhance provider ability in providing IUD services while at the same time having a positive impact on their reputation in local communities [19]. *Suraj* providers have previously identified that the role of female and male community mobilizers is of critical importance in mobilizing the community and increasing their FP clientele [19]. The impact on contraceptive use by MWRA in *Suraj* areas and specifically the significant increase in IUD use by MWRA in both the *Suraj* and CMW intervention arms is indicative of a need to adopt similar strategies for public contraceptive promotion programs. In addition both intervention models also demonstrated high IUD method continuation rates [16, 17], providing a strong rationale for scaling up of Suraj as well as CMW intervention at the national level to promote modern contraception. An earlier study also documented similar improvements in the IUD continuation rates at 12-months period (18.8 %) after using Suraj model as an intervention along with free vouchers which is significantly lower than the national trend of 26 % [18]. However, this will entail comprehensive training of not only the health care providers, but community based mobilizers as well who have direct access to potential clients in targeted communities. This finding from our study corroborated a 2002 national survey that married women living within 5km of community-based workers who have direct access to potential clients were significantly more likely to use modern reversible methods than those with no access [23]. The *Suraj* voucher scheme has the potential to have a national level impact on FP service uptake however, three key factors will determine the reach of the voucher program (i) keeping management costs low, (ii) inducing a large demand-side response among the two low socio-economic quintiles, and (iii) achieving a quality of care that translates a greater number of facility-based deliveries into a reduction in maternal morbidity and mortality [24].

In addition to training and capacity building, financial incentives are an important factor in encouraging women to adopt contraceptive methods [25]. The findings emphasize that approaches like *Suraj model*, when complemented with vouchers and community mobilization efforts, can improve the utilization of long-term contraceptive methods among rural and underserved women.

Evidence suggests that financial incentives can enhance demand, as well as impact the quality and quantity of maternal health services [25]. This is possible as financial incentives can be useful in overcoming health system and financial barriers that prevent women from accessing services and providers from delivering quality maternal care [24]. Vouchers deliver subsidies to individuals who otherwise would have to seek the services of an unskilled provider or most likely would not have sought care [26]. Social franchising complemented with targeted voucher schemes not only improves access to FP services but also helps reduce inequalities in health services and enables the extremely poor or financially vulnerable population groups to avail these services [27].

Overall, both *Suraj* and the CMW intervention models not only demonstrated increase in the use of long term contraceptive method – intra uterine device (IUDs) among the married women of reproductive age but based on a very recent evidence piece from a nested study from this same project confirms that both *Suraj* and CMW providers are similarly capable of ensuring higher IUD method continuation rate at different intervals [16, 17]. For example, at 12-month interval, the cumulative probability of IUD continuation in Suraj and CMW models were 85 % and 94 %, respectively; and likewise it was 82 and 80 % at 24 months. Such low discontinuation rates are well below the national average [5, 17]. Hence, it is proposed that both the government and private sectors may consider training the community midwives as well as to engage with the non-regulated private sector mid-level providers to promote the use of IUDs in Pakistan which presently is very low – 2.3 % [5].

The present findings somewhat also confirms the alarming need of trained and qualified female healthcare providers for long term reversible method of contraception at local health facilities instead of periodical fertility camps arranged by government or private sector [16]. This need was identified during a pre-project qualitative inquiry/needs assessment (QUAL 1a – refer to Fig. 1) by the general population – men and women of reproductive age residing in the similar project study areas/sites [27]. The project showed uptake and continuity of long term IUDs, with attempts to address access, affordability, availability about modern contraception. The project was also able to involve men as identified in the needs assessment in order to meet women and couples needs to fulfill their fertility and reproductive health objectives [27].

The results should be interpreted with caution. Quasi-experimental designs using pre and post intervention analysis can have some limitations. The study clients are not randomly assigned. However, pre-post intervention analysis with control is internationally accredited for use in situations where controlled trials are not feasible due to logistic, financial or other ethical reasons. This was a field project in a real life situation and due to the nature of the intervention i.e. vouchers and provision of contraceptive services made it difficult to blind the study participants.

We ensured that there was no spill over within different intervention areas by choosing areas at a minimum distance from each other. The difference in cultural background of participants from the intervention and control areas is a potential limitation. However, since the intervention and control areas are located within the same province we believe the differences would be minimal with a consequently limited impact on study findings. Another potential limitation is the presence of competing health providers, providing family planning services, operating within the areas of project health providers. Selecting a health care facility for the project where no other service providers exist is difficult. To address this limitation we had a control group to assess the impact of routine practice in health facilities towards family planning. Therefore, we are confident that the increase in outcomes in our study was due to the project intervention(s).

The findings of the study can be generalized to other settings with similar context. Besides taking into account the intervention details, replication will need to take into account the local cultural sensitivities as well as the local health system structure where the research is expected to be replicated.

## Conclusion

The successful implementation of *Suraj* intervention scheme highlights the importance of demand generation in tandem with provision of low cost family planning services embedded within the communities of the beneficiaries. The FP service integration with existing CMW providers approach also has some benefits in improving FP uptake (especially IUD) at the community level with increased probability of method continuation. Since the CMW interventions were not subsidized or free, the approach may be sustainable in the long term ensuring access to FP services for the underserved population segments. In addition, having dedicated and full-time community health workers or lady health workers (LHWs) for modern contraceptive services such as IUDs can facilitate connecting prospective and current users with the respective facility for building a strong referral system – either by increasing the existing LHWs numbers or introducing new cadre of FP field workers.

It will be beneficial to conduct further research on evaluating the FP integration approach in order to identify factors that can facilitate potential expansion of the approach in other areas of Pakistan with explicit focus on costing perspectives.

## Abbreviations

CMW: community midwife
DSF: demand-side financing
FCM: female community mobiliser
FP: family planning
IUD: intrauterine device
KP: Khyber Pakhtunkhwa
LARC: long-acting reversible contraceptives
MSS: Marie Stopes Society
MWRA: married women of reproductive age
TFR: total fertility rate
WHO: World Health Organization

## References

[1] Jain AK, Mahmood A, Sathar ZA, Masood I (2014). Reducing unmet need and unwanted childbearing: Evidence from a panel survey in Pakistan. Studies in Family Planning.

[2] World Population Data Sheet 2014 [Internet]. PRB. 2014. Available from: http://www.prb.org/pdf14/2014-world-populationdata-sheet_eng.pdf.

[3] Bhutta ZA, Hafeez A, Rizvi A, Ali N, Khan A, Ahmad F (2013). Reproductive, maternal, newborn, and child health in Pakistan: challenges and opportunities. The Lancet.

[4] Anderson BO, Yip CH, Ramsey SD, Bengoa R, Barun S (2006). Breast cancer in limited- resource countries: health care systems and public policy. Breast J.

[5] National Institute of Population Studies Pakistan, Macro International Inc (2014). Pakistan Demographic and Health Survey 2012–13.

[6] Bongaarts J, Mir AM, Mahmood A (2014). Policies for Capturing the Demographic Dividend in Pakistan.

[7] USAID (2012). What Unmet Need for Family Planning means in Pakistan.

[8] Word Development Indicators [Internet]. 2015 [cited December 2015]. Available from: http://data.worldbank.org/indicator/SP.DYN.CONU.ZS.

[9] WHO. Public policy and franchising reproductive health: current evidence and future directions. Geneva: World Health Organization, Department of Reproductive Health and Research; 2007. ISBN: 978 92 4 159602 1. Available from: http://apps.who.int/iris/bitstream/10665/43735/1/9789241596021_eng.pdf.

[10] Azmat SK, Shaikh BT, Hameed W, Mustafa G, Hussain W, Asghar J (2013). Impact of social franchising on contraceptive use when complemented by vouchers: a quasi- experimental study in Rural Pakistan. PLoS One.

[11] Murray S, Hunter B, Bisht R, Ensor T, Bick D (2014). Effects of demand-side financing on utilisation, experiences and outcomes of maternity care in low- and middle-income countries: a systematic review. BMC Pregnancy Childbirth.

[12] Sarfraz M, Hamid S (2014). Challenges in delivery of skilled maternal care - experiences of community midwives in Pakistan. BMC pregnancy and childbirth..

[13] Maternal and Newborn Health Programme Research and Advocacy Fund, 2015, DIFD; [cited 2015 December 15]. Available from: http://r4d.dfid.gov.uk/Project/60901/Default.aspx

[14] MSS. RAF “EVIDENCE FOR INNOVATING TO SAVE LIVES” Karachi: Marie Stopes Society Pakistan; 2014 [cited 2015 December 15]. ‘Evidence for Innovating to Save Lives’ is a project funded by the Maternal and Newborn Health Programme – Research and Advocacy Fund (RAF) British Council Pakistan jointly funded by DFID and AusAID, and is implemented by Marie Stopes Society (MSS)]. Available from: http://mariestopespk.org/raf/research-project/

[15] RAF, Quarterly Newsletter, Issue 7, January-March 2014 Islamabad: Maternal and Newborn Health Programme Research and Advocacy Fund (RAF), British Council Pakistan; [cited 2015 December 15]. Available from: http://r4d.dfid.gov.uk/pdf/outputs/RAF/RAF_Newsletter_Issue7.pdf

[16] Hirose A, Hall S, Memon Z, Hussein J (2015). Bridging evidence, policy, and practice to strengthen health systems for improved maternal and newborn health in Pakistan. Health Res Policy Syst.

[17] Hameed W, Azmat SK, Ishaque M, Hussain W, Mustafa G, Khan OF (2015). Continuation rates and reasons for discontinuation of intra-uterine device in three provinces of Pakistan: results of a 24-month prospective client follow-up. Health Res Policy Syst.

[18] Azmat SK, Shaikh BT, Hameed W, Bilgrami M, Mustafa G, Ali M (2012). Rates of IUCD discontinuation and its associated factors among the clients of a social franchising network in Pakistan. BMC Women's Health.

[19] Azmat SK, Mustafa G, Hameed W, Asghar J, Ahmed A, Shaikh BT (2013). Social franchising and vouchers to promote long-term methods of family planning in rural pakistan: a qualitative stocktaking with stakeholders. J Pak Med Assoc.

[20] Najafi-Sharjabad F, Yahya S, Zainiyah S, Abdul Rahman H, Hanafiah M (2013). Barriers of modern contraceptive practices among Asian women: A mini literature review. Global J Health Sci.

[21] Pearson M (2001). Demand Side Financing for Health Care.

[22] Nishtar NA, Sami N, Alim S, Pradhan N, Hasnain FU (2013). Determinants of contraceptives use amongst youth: an exploratory study with family planning service providers in Karachi Pakistan. Global J Health Sci.

[23] Sultan M, Cleland JG, Ali MM (2002). Assessment of a new approach to family planning services in rural Pakistan. Am J Public Health.

[24] Bellows BW, Conlon CM, Higges ES, Townsend JW, Nahed MG, Cavanaugh K (2013). A taxonomy and results from a comprehensive review of 28 maternal health voucher programs. J Health Popul Nutr.

[25] Morgan L, Staton ME, Higgs ES, Blaster RL, Bellows BW, Brandes N (2013). Financial incentives and maternal health: where do we go from here?. J Health Popul Nutr.

[26] Grainger C, Gorter A, Okal J, Bellows B (2014). Lessons from sexual and reproductive health voucher program design and function: a comprehensive review. Int J Equity Health..

[27] Mustafa G, Azmat SK, Hameed W, Ali S, Ishaque M, Hussain W, Ahmed A and Munroe E. Family Planning Knowledge, Attitudes, and Practices among Married Men and Women in Rural Areas of Pakistan: Findings from a Qualitative Need Assessment Study. Inter J Reproduct Med; 2015:190520. doi:10.1155/2015/190520 10.1155/2015/190520 PMC4569791 26421316

